# Webster's New Unabridged Dictionary

**Published:** 1881-02

**Authors:** 


### BIBLIOGRAPHICAL.

---

*Webster's New Unabridged Dictionary.*—

The man who said that "the place to find consolation was in the dictionary," was right, and we find, after years of work of a literary kind, more or less severe, and laboring under peculiar disadvantages in writing, that our best place to find words is in our old copy of Webster's Unabridged. It is a compendium of knowledge, so vast and universal as to be indeed nigh wonderful; and we see with surprise that the publishers, Messrs. Merriom, Springfield, Mass., are issuing a new edition, and learn further that in addition to the 118,000 words in the former, this edition has 4,600 new words, besides a biographical dictionary of 9,700 names. Any one who wishes a book, good for all time will find it here. We confess that we often turn from Appleton's and Johnson's cyclopædias and find in Webster's dictionary, what we vainly looked for in their ponderous volumes. It is truly a great work. H.

